# Visual Outcomes of Anti-VEGF Treatment on Neovascular Age-Related Macular Degeneration: A Real-World Population-Based Cohort Study

**DOI:** 10.3390/ph16070927

**Published:** 2023-06-26

**Authors:** Ida Korva-Gurung, Anna-Maria Kubin, Pasi Ohtonen, Nina Hautala

**Affiliations:** 1 Research Unit of Clinical Medicine and Medical Research Center, Department of Ophthalmology, Oulu University Hospital and University of Oulu, 90029 Oulu, Finland; ida.korva-gurung@student.oulu.fi (I.K.-G.); anna-maria.kubin@oulu.fi (A.-M.K.); 2 Medical Research Center, PEDEGO Research Unit, Department of Ophthalmology, Oulu University Hospital and University of Oulu, 90029 Oulu, Finland; 3 Research Service Unit, 90220 Oulu, Finland; pasi.ohtonen@oulu.fi; 4 The Research Unit of Surgery, Anesthesia and Intensive Care, Oulu University Hospital and University of Oulu, 90029 Oulu, Finland

**Keywords:** neovascular age-related macular degeneration, visual outcomes, anti-VEGF, visual impairment, population-based cohort

## Abstract

Neovascular age-related macular degeneration (nAMD) leads to visual impairment if not treated promptly. Intravitreal anti-VEGF drugs have revolutionized nAMD treatment in the past two decades. We evaluated the visual outcomes of anti-VEGF treatment in nAMD. A real-life population-based cohort study. The data included parameters for age, sex, age at diagnosis, laterality, chronicity, symptoms, visual outcomes, lens status, and history of intravitreal injections. A total of 1088 eyes (827 patients) with nAMD were included. Visual acuity was stable or improved in 984 eyes (90%) after an average of 36 ± 25 months of follow-up. Bevacizumab was the first-line drug in 1083 (99.5%) eyes. Vision improved ≥15 ETDRS letters in 377 (35%), >5 ETDRS letters in 309 (28%), and was stable (±5 ETDRS letters) in 298 (27%) eyes after anti-VEGF treatment. The loss of 5 ≤ 15 ETDRS letters in 44 (4%) eyes and ≥15 ETDRS letters in 60 (6%) eyes was noted. At the diagnosis of nAMD, 110 out of 827 patients (13%) fulfilled the criteria for visual impairment, whereas 179 patients (22%) were visually impaired after the follow-up. Improvement or stabilization in vision was noted in 90% of the anti-VEGF-treated eyes with nAMD. In addition, anti-VEGF agents are crucial in diminishing nAMD-related visual impairment.

## 1. Introduction

Age-related macular degeneration (AMD) is the most common cause of severe visual impairment in industrialized countries [1,2]. Especially the late forms of the disease, neovascular AMD (nAMD) and geographic atrophy, are responsible for the most visual loss [3]. AMD is an increasingly important public health challenge worldwide due to the increase in aging populations and lifetime expectancy. Accordingly, the prevalence of AMD has been projected to rise from 196 million in 2020 to 288 million by the year 2040 [4]. 

AMD risk is influenced by genetic and non-genetic factors [5,6,7,8]. The exact pathogenesis of AMD is not fully understood, but it is thought to result from multifactorial processes [9,10,11]. During the normal retinal ageing processes, cumulative oxidative damage contributes to anatomical and physiological changes at the level of photoreceptors, the retinal pigment epithelium (RPE), Bruch’s membrane, and the choriocapillaris. In the pathogenesis of AMD, thickening of the RPE-Bruch’s membrane complex and decreased circulation may lead to the accumulation of lipofuscin in RPE cells. Pigmentary abnormalities of the RPE are also typical. Complement system activation may lead to the degeneration of the choriocapillaris, which may further cause oxidative injury to the RPE, promote inflammation, and finally lead to the death of the photoreceptor cells in the central macular area. A hallmark of nAMD, choroidal neovascularization, may develop after a breakdown of Bruch’s membrane [9,10,11]. The previous systematic review and meta-analysis by Chakravarthy et al. identified four strong and consistent risk factors for the development of late AMD, including age, smoking, previous cataract surgery, and a family history of AMD. Additional risk factors associated with AMD were obesity, a history of cardiovascular disease, hypertension, and plasma fibrinogen concentration [5]. The association of very rare coding variants in complement factor H (CFH), complement factor I (CFI), and tissue inhibitor of metalloproteinases-3 (TIMP3) has suggested causal roles for these genes in AMD pathogenesis [6].

AMD is clinically divided into early, intermediate, and late forms [1,2]. Early AMD is defined as the presence of medium-sized drusen without retinal hyper- or hypo-pigmentary changes in the macula. The presence of at least one large drusen (>125 µm) or extensive medium drusen and typical pigmentary changes are typical for intermediate AMD. Early and intermediate stages of AMD are often asymptomatic or associated with only mild central distortion. Both nAMD and atrophic AMD are defined as late AMD, causing typical symptoms including reduction of visual acuity, distorted central vision, and the development of a central scotoma [1]. The symptoms and clinical findings of nAMD may progress rapidly over weeks or months, while atrophic AMD progresses more slowly over years or decades. 

The current treatment protocols for AMD are focused on the management of nAMD since there are no commonly available treatment strategies for atrophic AMD at present [1,2,12], although the complement C3 inhibitor pegcetacoplan has recently been reported to reduce the growth of geographic atrophy in patients with dry AMD [13,14]. The pathogenesis of nAMD involves the development of choroidal neovascularization in the macula, vascular leakage leading to macular edema, hemorrhages, and occasionally scarring, which may lead to permanent loss of vision [15,16]. Several mediators are shown to have a role in this complex pathological process, including kinases, cytokines, and growth factors [1,2,17,18]. Vascular endothelial growth factor A (VEGF-A) and its receptors have the notable ability to promote angiogenesis and vascular permeability, which explains their significance in the management of nAMD [18]. Over the past two decades, the use of VEGF-inhibitors such as ranibizumab, bevacizumab, aflibercept, brolucizumab, and faricimab has revolutionized the clinical management of nAMD [1,2,12,19,20,21]. 

The current population-based study aimed to evaluate the visual outcomes of anti-VEGF treatment for nAMD in a real-life setting. In addition, the rates of visual impairment were determined at the onset of nAMD and after the intravitreal anti-VEGF treatment. Our results suggest that a great majority of individuals with nAMD, 90%, benefit from anti-VEGF management in terms of stabilization or improvement of visual acuity. 

## 2. Results

### 2.1. Characteristics of the Study Population

A total of 827 nAMD patients with 1088 eyes were included in the study. All study eyes had nAMD and were treated with intravitreal anti-VEGF injections. The average age of the nAMD patients at the time of the diagnosis was 78 ± 8 years, and 693 out of 827 patients (64%) were women. The study patients were followed up for 36 ± 25 months (range 3–134 months) on average. The eyes with the most ETDRS letters gained were followed up longer than those with the biggest loss of vision (40 ± 25 vs. 20 ± 20 months, respectively, *p* < 0.001) (Table 1). 

Bilateral nAMD was diagnosed in 107 (13%) patients already at the onset of nAMD.

During the follow-up period, 154 patients (19%) developed nAMD also in the other-eye at an average time interval of 14 ± 19 months after the initial diagnosis of nAMD. At the end of the follow-up, a total of 261 (32%) patients with nAMD had bilateral disease. 

The duration of typical visual symptoms of nAMD before diagnosis was less than two months in 36% of the patients and over two months in 64% of the patients. Improvement in visual acuity >5 ETDRS letters was noted in 69% of the patients with the duration of ocular symptoms of nAMD for less than two months and in 62% with symptoms longer than two months (*p* = 0.211). In both groups, 9% of the patients lost >5 ETDRS letters. The symptoms complained of were a decrease in visual acuity (76%), distortion of straight lines (34%), blurring of the central vision (19%), and other discomfort of the eyes (2%). Several subsequent symptoms occurred commonly in study participants with nAMD. 

### 2.2. Visual Outcomes

**Figure 1:**
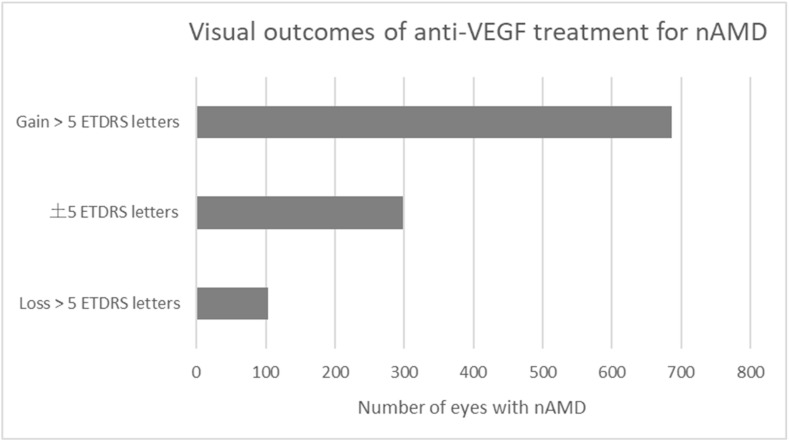
Visual outcomes of intravitreal anti-VEGF treatment for nAMD.

An average baseline visual acuity was 56 ± 20 ETDRS letters. During the follow-up period, visual acuity was stable or improved in 984 (90%) eyes. The gain of >5 ETDRS letters was achieved in 686 (63%) of the total of 1088 study eyes with nAMD (Figure 1). In this visual outcome group, ≥15 ETDRS letter gain was noted in 377 (35%) eyes. Visual acuity remained stable (±5 ETDRS letters) in 298 (27%) eyes (Figure 1). The loss of >5 ETDRS letters was noted in 104 (10%) eyes, which includes 60 (6%) eyes with a loss of ≥15 ETDRS letters (Figure 1). The average age of the patients in each visual outcome group at the onset of nAMD ranged from 77 to 80 (*p* = 0.022). In addition, female gender was represented in 63–64% of the patients in all visual outcome groups (*p* = 0.951). 

### 2.3. Intravitreal Anti-VEGF Injections

The number of anti-VEGF injections varied in different visual outcome groups. An average number of anti-VEGF injections was 8 ± 10, 15 ± 14, and 19 ± 15 in the groups of loss >5 ETDRS letters, no change in vision, or gain >5 ETDRS letters, respectively (*p* < 0.001) (Table 1). A total of 45% of the eyes received less than ten injections, and 81% received less than 30 injections during the follow-up period. Only single eyes were treated with over 50 injections, and the eye with the most injections received 80 injections during the study period. An average number of injections per eye was 17 ± 15 in the whole study population during the follow-up.

The time interval between anti-VEGF injections ranged from 28 to 2117 days. 695 (64%) eyes had chronic nAMD and needed continuous intravitreal treatment during the follow-up period. Intravitreal anti-VEGF treatment breaks varying from 4 to 71 months were noted in 393 (36%) study eyes (*p* < 0.001) (Table 1). There were several reasons for the discontinuation of anti-VEGF injections during the follow-up: no need (dry macula) in 52%, no fulfillment of the treatment criteria (visual acuity > 0.05 in the Snellen E chart and sufficient compliance for treatment) in 20%, or no response to anti-VEGF treatment in 10% of the eyes. The patient asked for discontinuation of the injections, and in 9% of the cases, the reason for stopping the treatment was the death of the patient. No information about the reason for the discontinuation of the treatment was available in 1% of the eyes.

Remission of active nAMD (>4 months break with no need for anti-VEGF treatment) was noted in 42% of the eyes gaining most vision and in 31% of those with stable visual activity (Table 1). In contrast, only 14% of the eyes with a loss of vision emerged without continuous treatment. 

Bevacizumab was the first-line drug in 1083 (99.5%) eyes, whereas 5 eyes (0.5%) were initially treated with aflibercept. Endophthalmitis occurred in 11 out of a total of 18,359 injections (0.06%) during the study period.

### 2.4. The Impact of Cataract

**Figure 2:**
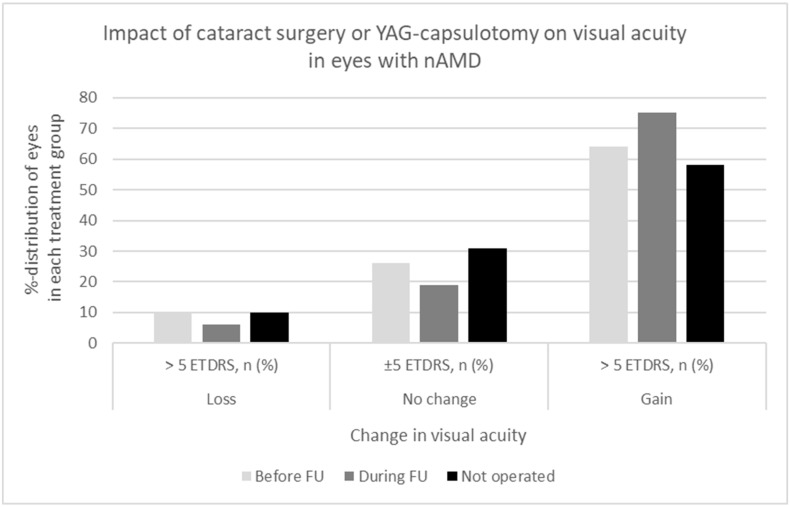
Impact of cataract surgery or YAG capsulotomy on visual acuity in eyes with nAMD.

The impact of the lens status (pseudophakic, phakic), cataract, secondary cataract, cataract surgery, and neodymium-doped yttrium aluminum garnet laser capsulotomy (YAG-capsulotomy) on visual acuity was evaluated according to the relatively old age of the study patients and the long follow-up period. Cataract surgery, or YAG-capsulotomy, had been completed in 428 (39%) eyes before the diagnosis of nAMD. In 162 (15%) eyes, either cataract or secondary cataract were operated on during the follow-up of nAMD, and 498 (46%) eyes remained phakic until the end of the follow-up period. The effect of cataract surgery, YAG-capsulotomy, or developing cataracts on visual acuity in eyes with nAMD is shown in detail in Figure 2. Visual acuity improved >5 ETDRS letters in 64% of the eyes in the pseudophakic group, in 75% of the eyes in the group of cataract surgery/YAG-capsulotomy during the follow-up, and in 58% of the eyes in the group without any intervention for cataract (*p* = 0.003).

### 2.5. Visual Impairment

At the time of the diagnosis of nAMD, 110 out of 827 patients (13%) fulfilled the criteria for visual impairment, whereas a total of 179 patients (22%) were visually impaired after the follow-up. However, 28 (25%) patients with initial vision impairment were not visually impaired at the end of follow-up, and 97 out of 717 (14%) patients not visually impaired at the diagnosis of nAMD fulfilled the criteria at the end of the follow-up period.

## 3. Discussion

Intravitreal anti-VEGF therapy is the standard of care for the treatment of nAMD [20]. Currently available therapies include aflibercept, bevacizumab, brolucizumab, faricimab, and ranibizumab [19,22,23,24,25]. Landmark trials for anti-VEGF agents have proven major advancements in the management and prognosis of nAMD, which is known to lead to permanently impaired vision over time if not treated [26,27,28,29,30]. However, clinical trial treatment regimens may not reflect real-world practice, and the high frequency of intravitreal anti-VEGF injections in registration trial designs may lead to a high treatment burden and affect the outcomes. The present study was designed to expose the real-life treatment outcomes of nAMD in a population-based cohort during a long follow-up. 

The study nAMD cohort is comparable to the recent study of 67,666 eyes treated with intravitreal anti-VEGF agents for nAMD according to the age of the patients, sex distribution, and baseline visual acuity [31]. Ciulla et al. reported an improvement in visual acuity of 3 ETDRS letters during the first year of intravitreal treatment for nAMD, followed by a gradual loss of vision back to baseline over the next 4 years [31]. Similarly, several clinical extension studies of randomized nAMD trials have reported that visual acuity commonly declines over the years after an increase at the start of the anti-VEGF treatment [32,33,34]. These studies revealed a mean visual gain of only 2 ETDRS letters by treatment year 4 and a loss of 3 and 9 letters by 5.5 and 7 years, respectively. Our results show stable or improved visual acuity in 90% of the eyes during follow-up for 3 years on average. This is in line with the recent study that revealed stable or >10 ETDRS letter improvement in visual acuity by brolucizumab in 84% of treatment-naïve and 86% of previously treated eyes with nAMD [35]. In the current study, losses of >5 ETDRS letters were noted in 10% of all eyes. Chandra et al. reported a 15-letter decline in visual acuity in 24% of eyes with nAMD, mostly caused by macular atrophy [36]. These differences in visual outcomes can possibly be explained by differences in the rates of macular atrophy in the study cohorts as well as the duration and frequency of the anti-VEGF injections. 

A recent registry study documented that over one-third of the patients with nAMD had discontinued intravitreal anti-VEGF treatment by the end of the first year [37]. Continuous and frequent treatment triggers a remarkable load not only for healthcare providers but also on patients, although the injection frequency may decline over the years [38]. In our cohort, intravitreal anti-VEGF-treatment was discontinued in cases of dry macula with no intra- or subretinal fluid observed, treatment resistance, or if the patients requested discontinuation. Anti-VEGF injections were restarted in a case of renewed active disease. Despite almost 6-year breaks in anti-VEGF treatment in the inactive phase of the disease in some individuals, no reactivation of nAMD nor reduced visual acuity were noted after the follow-up. One may assume that marked cost savings can be achieved by active follow-up of discontinued patient cohorts when compared to continuous and frequent injections in all patients. In this cohort of 1088 eyes, only a part, 64%, needed continuous anti-VEGF treatment for active nAMD. Some previous studies have shown that interruption of the regular treatment may have caused at least a transient worsening of visual acuity, mostly due to the development of macular hemorrhages [39,40,41,42]. It seems possible that progression of nAMD may have occurred if the interruptions of injections were implemented while the disease was still active with choroidal neovascularization. Nassisi et al. reported no significant differences in visual outcomes in patients with a delay in anti-VEGF injections for a few months due to COVID-19 lockdown and no development of atrophy or fibrosis at 6 months [43].

Bevacizumab was the first-line drug in almost all (99.5%) of the study eyes, which is in accordance with the Finnish Current Care Guidelines [12]. Similarly, in a recent study by Nassisi et al., over 95% of the individuals with nAMD were treated with bevacizumab [43]. Ross et al. compared the cost-effectiveness of aflibercept, bevacizumab, and ranibizumab for the treatment of diabetic macular edema. Aflibercept and ranibizumab were found to be 31 and 20 times more expensive than bevacizumab, respectively [44]. Off-label use of bevacizumab in the management of both nAMD and diabetic macular edema has become commonly available throughout Europe and the US because its efficacy and safety compare to those of other anti-VEGF agents at lower costs [33,45,46]. Recurrent intravitreal injections, typically at 4–8 week intervals, for an increasing aging population with nAMD impose a high financial burden on health care. The eyes in the present study received on average 17 anti-VEGF injections during the follow-up, and the number of injections increased along with the number of letters gained. Recently, the bispecific antibody faricimab, which acts via dual inhibition of both VEGF-A and angiopoietin-2, has shown the possibility of extending the injection intervals up to 16 weeks with sustained efficacy for nAMD [30]. Fewer intravitreal treatments with longer intervals between treatments would be helpful for reducing the burden and costs for both patients and health care providers.

Age-related cataracts often coexist with AMD. The impact of cataract or secondary cataract was considered as the visual outcomes were analyzed. Pseudophakia has been found to be associated with better visual outcomes compared to phakic eyes [47,48]. In accordance, our results showed that approximately 60% of the patients with the highest ≥15 ETDRS letter increase in visual acuity had undergone cataract surgery before or during the follow-up. In addition, after cataract surgery completed before or during the follow-up, 64% and 75% of the eyes, respectively, had a gain of >5 ETDRS letters compared to 58% in the phakic eyes. However, a marked increase in visual acuity was also noted in phakic eyes, which may suggest that not all individuals develop visually significant cataracts and that progressing cataracts are likely to be treated timely in Finland according to the Finnish current care guidelines for cataract [49]. Cataract surgery has been shown not to have a clinically significant impact on the activity of pre-existing choroidal neovascularization secondary to nAMD and may be recommended for patients with nAMD and cataracts that limit vision [50].

Anti-VEGF agents have reduced visual impairment due to nAMD and improved patients’ quality of life [2,51,52]. In our study, the number of patients with visual impairment increased during the 10-year period, which reflects the natural course of the disease. It is, however, notable that one fourth of the patients with initial visual impairment benefited from anti-VEGF treatment and were no longer visually impaired at the end of the follow-up. Despite treatment, 14% of the study patients had lost their visual acuity to the level of visual impairment by the end of the study period. Poor visual gain among patients with longstanding symptoms suggests that timely diagnosis and treatment of nAMD are prudent, and the delay in the initiation of treatment may have a negative impact on visual outcomes. 

The study has some limitations. There are variations in the duration of follow-up, amount of anti-VEGF injections, and implementation of treatment due to the real-life setting and retrospective nature of the study. The administration of anti-VEGF agents in various treatment regimens may vary and thus affect the treatment outcomes and the number of injections. In most cases, treatment started with monthly injections with anti-VEGF agents and continued according to the treatment response of the individual patient. The real-life setting might be considered a strength of the present study, as might a population-based cohort of 827 patients with 1088 eyes with nAMD in a long-term follow-up period. The implementation of the treatment and the results in the randomized, controlled studies may differ from those obtained in the real-world setting. In addition, the present study cohort includes only patients with nAMD, in contrast to numerous studies that include patients with any AMD.

## 4. Materials and Methods

### 4.1. Characterization of the Study Population and Environment

The population-based cohort consisted of patients with neovascular age-related macular degeneration (nAMD) diagnosed in the Northern Ostrobothnia Hospital District in 2010–2016. The data from patient records was collected until 2019 to ensure a sufficient duration of follow-up for all patients. The treatment of nAMD was completed at Oulu University Hospital, which is responsible for tertiary care for a population of approximately 410,000 inhabitants. According to the data from Statistics Finland, over 98% of the population aged over 75 years in Finland is of Finnish background (white Caucasian by ethnicity). In Finland, municipalities are responsible for organizing and financing health care, and every citizen is entitled to adequate health services. Specialized healthcare refers to examinations and treatments arranged in hospitals in specialized fields, such as ophthalmological examinations and treatment of eye diseases, including nAMD. Access to ophthalmic treatment requires a referral.

### 4.2. Diagnostic Evaluations for nAMD and Exclusion Criteria

The hospital’s electronic patient database was used to search for the nAMD patients treated with intravitreal anti-VEGF agents by using the ICD-10 (International Classification of Diseases) diagnosis code for nAMD (H35.31). All patients with a nAMD diagnosis had undergone a comprehensive ophthalmic examination, evaluation of best-corrected visual acuity, and fundus imaging (fundus photography, fluorescein angiogram, optical coherence tomography (OCT), and optical coherence tomography angiography (OCT-A)) based on the discretion of the treating physician and the availability of the imaging techniques during the follow-up period. The cases of dry AMD, polypoidal choroidal vasculopathy (PCV), myopic degeneration, or other retinal disorders, such as diabetic macular edema, treated with anti-VEGF injections were excluded from the study. Patients with less than three consecutive anti-VEGF injections, a follow-up time less than 3 months, or a diagnosis of dry AMD were excluded from the study. 

### 4.3. Treatment Regimens for nAMD

The patients with nAMD were treated following the Pro re nata (PRN) or treat and extend (T&E) regimens for nAMD following the Finnish current care guidelines for age-related macular degeneration [12]. Generally, the first 3–6 injections were scheduled at 4–6 week intervals, and the response to treatment was assessed by evaluation of visual acuity, OCT, and fundus photography. Intra- and subretinal fluid and macular hemorrhages were indications to continue anti-VEGF treatment. In cases of dry macula, the patients were followed up at 1–2-month intervals to diagnose the possible renewal of macular edema. In Finland, bevacizumab is commonly the first-line choice for the treatment of nAMD.

### 4.4. Clinical Outcome Measures

Demographic and clinical data of the patients was collected and included parameters for age, gender, age at diagnosis of nAMD, duration of ocular symptoms, time of follow-up, laterality, chronicity, best-corrected visual acuity, and the rate of visual impairment at baseline and during follow-up, the number of intravitreal injections, lens status (phakic or pseudophakic), and rates for cataract surgery or YAG-capsulotomy. The intravitreal anti-VEGF treatment was considered continuous if the longest interval between the two injections was less than 4 months during the follow-up. The impact of lens status on visual acuity was observed according to the relatively old age of the cohort patients and the long follow-up period. The comprehensive ophthalmic evaluations, macularOCT, and fundus photography were completed at the onset and repeatedly during the follow-up period. Visual acuity was evaluated routinely by the Snellen E-chart, and the decimals were converted to ETDRS letters for the evaluation of changes in visual acuity. The WHO criteria for distance vision impairment (Snellen visual acuity worse than 0.3 or ETDRS less than 59 letters in a better eye) were used.

### 4.5. Ethical Aspects of the Study

The study followed the tenets of the Declaration of Helsinki, and it was conducted with the approval of the Oulu University Hospital Research Committee (221/2016). A written informed consent was obtained from the participants at the time of the clinical ophthalmic evaluation.

### 4.6. Statistical Analysis

SPSS for Windows (IBM Corp., Released 2019). IBM SPSS Statistics for Windows, Version 26.0. (Armonk, NY, USA: IBM Corp.) was used for data analysis. Between-group comparisons for continuous variables were performed using the analysis of variance or Brown–Forsythe test, the latter if the homogeneity of variances test failed. Categorical data was analyzed using the Pearson Chi-squared test.

## 5. Conclusions

These real-life results show that a great majority of patients with nAMD benefit from intravitreal anti-VEGF treatment in terms of stability or improvement in visual acuity.

Bevacizumab seems to be an effective and safe treatment for nAMD with relatively low costs.

The results of this study suggest that continuous anti-VEGF injections are not necessary in cases of dry macula after treatment because several nAMD patients had long, even several-year intervals without frequent injections and still showed no decline in long-term visual outcomes. This should be considered when the burden for nAMD treatment increases along with an increase in the number of aging populations.

## Figures and Tables

**Table 1:** The distribution of the study eyes with nAMD, duration of follow-up, number and interval of intravitreal anti-VEGF injections, and number of eyes with over four-month break in anti-VEGF treatment in different visual outcome groups.

	Change in Visual Acuity in ETDRS Letters			
	Loss >5 ETDRS (*n* = 104)	No Change ±5 ETDRS (*n* = 298)	Gain >5 ETDRS (*n* = 686)	*p*-Value
Number of eyes, *n* (%)	104 (10)	298 (27)	686 (63)	
Time of follow-up, mean (SD)	20 (20)	31 (23)	40 (25)	*p* < 0.001 ^1^
[min–max], months	[3–102]	[4–99]	[3–80]	
Number of anti-VEGF injections,				*p* < 0.001 ^1^
mean (SD)	8 (10)	15 (14)	19 (15)	
[min–max]	[3–75]	[3–71]	[3–80]	
Time interval between injections,				*p* = 0.003 ^1^
mean, days (SD)	120 (243)	161 (216)	197 (255)	
[min–max]	[28–1878]	[29–1442]	[28–2117]	
Remission of nAMD > 4 months, *n* (%)	15 (14)	93 (31)	285 (42)	*p* < 0.001 ^2^

^1^ Brown-Forsythe test, ^2^ Chi-square test.

## Data Availability

The data presented in this study are available on request from the corresponding author.
